# Impact of the COVID-19 pandemic on remote mental healthcare and prescribing in psychiatry

**DOI:** 10.1192/j.eurpsy.2023.199

**Published:** 2023-07-19

**Authors:** R. Patel

**Affiliations:** Institute of Psychiatry, Psychology & Neuroscience, King’s College London, London, United Kingdom

## Abstract

**Abstract:**

During the COVID-19 pandemic, mental health services often used remote technology to deliver care. However, the rise in remote consultation was largely unplanned. Furthermore, while many patients found remote technology a useful way to access care, others did not. Going forwards, remote technology will continue to play an important role in the delivery of mental healthcare. Research on the most effective ways for mental health services to implement remote technology is thus urgently needed.

Electronic health records (EHRs) have been widely adopted in mental healthcare services. EHRs not only support individual patient care, but also open the door to largescale research through the analysis of de-identified clinical data. The South London and Maudsley (SLaM) Biomedical Research Centre (BRC) Case Register is a large EHR dataset comprising structured and unstructured clinical information on patients receiving specialist mental healthcare. In this talk, I will present findings from an analysis of the SLaM BRC Case Register using the Clinical Record Interactive Search tool (CRIS) to evaluate the uptake of remote mental healthcare during the COVID-19 pandemic and its association with medication prescribing. I will discuss the implications of these findings for psychiatrists delivering community mental healthcare and how mental health services can harness remote technology to optimise clinical outcomes.

**Disclosure of Interest:**

R. Patel Grant / Research support from: NIHR (NIHR301690); MRC (MR/S003118/1); Academy of Medical Sciences (SGL015/1020); Janssen, Consultant of: Induction Healthcare Ltd; Holmusk

